# Risk profiles for smoke behavior in COVID-19: a classification and regression tree analysis approach

**DOI:** 10.1186/s12889-023-17224-z

**Published:** 2023-11-21

**Authors:** Jiangyun Chen, Jiao Yang, Siyuan Liu, Haozheng Zhou, Xuanhao Yin, Menglin Luo, Yibo Wu, Jinghui Chang

**Affiliations:** 1 School of Health Management, Southern Medical University, No.1023-1063 Shatai Road, Baiyun District, Guangzhou City, Guangdong Province China; 2 Institute of Health Management, Southern Medical University, No.1023-1063 Shatai Road, Baiyun District, Guangzhou City, Guangdong Province China; 3 Institute for Hospital Management of Henan Province, No. 1, Longhu Middle Ring Road, Jinshui District, Zhengzhou City, Henan Province China; 4 School of Public Health, Capital Medical University, 10 Xitoutiao, Youanmen, Beijing, China; 5 School of Public Health, Southern Medical University, No.1023-1063 Shatai Road, Baiyun District, Guangzhou City, Guangdong Province China; 6 School of Pharmaceutical, Southern Medical University, Guangzhou, China; 7 School of Public Health, Peking University, No.38 Xueyuan Road, Haidian District, Beijing City, China

**Keywords:** Classification and regression tree (CART), COVID-19, Smoking behavior

## Abstract

**Background:**

COVID-19 pandemic emerged worldwide at the end of 2019, causing a severe global public health threat, and smoking is closely related to COVID-19. Previous studies have reported changes in smoking behavior and influencing factors during the COVID-19 period, but none of them explored the main influencing factor and high-risk populations for smoking behavior during this period.

**Methods:**

We conducted a nationwide survey and obtained 21,916 valid data. Logistic regression was used to examine the relationships between each potential influencing factor (sociodemographic characteristics, perceived social support, depression, anxiety, and self-efficacy) and smoking outcomes. Then, variables related to smoking behavior were included based on the results of the multiple logistic regression, and the classification and regression tree (CART) method was used to determine the high-risk population for increased smoking behavior during COVID-19 and the most profound influencing factors on smoking increase. Finally, we used accuracy to evaluated the performance of the tree.

**Results:**

The strongest predictor of smoking behavior during the COVID-19 period is acceptance degree of passive smoking. The subgroup with a high acceptation degree of passive smoking, have no smokers smoked around, and a length of smoking of ≥ 30 years is identified as the highest smoking risk (34%). The accuracy of classification and regression tree is 87%.

**Conclusion:**

The main influencing factor is acceptance degree of passive smoking. More knowledge about the harm of secondhand smoke should be promoted. For high-risk population who smoke, the “mask protection” effect during the COVID-19 pandemic should be fully utilized to encourage smoking cessation.

**Supplementary Information:**

The online version contains supplementary material available at 10.1186/s12889-023-17224-z.

## Background

At the end of 2019, COVID-19 spread globally. In March 2020, WHO declared it a pandemic [1], which has led to significant years of life loss [2].and excess mortality [3]. Smoking is a closely related factor to COVID-19. On the one hand, smoking has been shown to upregulate ACE2 expression, increasing susceptibility to COVID-19 [4]. On the other hand, COVID-19 severity is significantly higher in smokers compared to non-smokers [5]. Therefore, it is necessary to reduce smoking behavior to promote health during the COVID-19 outbreak.

Some previous studies have reported changes in smoking behavior during the COVID-19 pandemic and identified influencing factors. Some studies suggest that smoking behavior has decreased during the pandemic due to concerns about the perceived harm of smoking during COVID-19 [6], difficulties purchasing cigarettes due to pandemic-related lockdowns, and the inability to smoke in public places due to mask-wearing requirements [7]. However, other studies have indicated a significant increase in smoking behavior during COVID-19 due to anxiety, depression, stress, and other factors [8, 9]. The multitude of factors influencing smoking during COVID-19 necessitates identifying high-risk populations and targeting the most significant influencing factors to reduce smoking behavior. However, none of these studies have investigated the primary influencing factors and high-risk populations for smoking behavior during the COVID-19 pandemic.

Classification and Regression Tree Analysis (CART) is a decision tree method developed by Breiman and colleagues. Using CART, it is possible to identify the most significant influencing factors for relative risk and explore the interaction between influencing factors and the most critical influencing factors to form the branches of the classification and regression tree, dividing the population into high-risk subgroups [10]. It is a nonparametric program that begins tree development by examining all predictor variables and selecting the variable (parent node) that can best predict the desired classification. The data in this parent node is divided into two classifications (child nodes): one predicts the response variable classification, and the other does not. This binary recursive splitting process is repeated for each child node until further splitting is no longer possible [11]. Over the years, as CART has developed, it has been increasingly used in the medical field [12–15], and in the smoking field, it is mainly used to identify high-risk populations for the use of tobacco substitutes [16], the combination of risk factors for smoking and the strongest predictive indicators [17], as well as the prediction of smoking cessation outcomes [18].

### The present study

Overall, smoking is a risk factor for COVID-19 infection and severity. Prior studies have analyzed the influencing factors of smoking behavior during the pandemic, but these studies only explored the relationship between influencing factors and changes in smoking behavior. This study aims to address the limitations of these studies. Specifically, CART analysis was used to explore the factors that most deeply influence smoking behavior in the population and to analyze the interactions between this factor and other influencing factors to identify high-risk populations for increased smoking behavior.

## Method

### Data and procedure

The data used in this study is conducted in 23 provinces, 5 autonomous regions, and 4 municipalities directly under the central government from June 20, 2022, to August 31, 2022. In this time, China was still experiencing the peak of the COVID-19 pandemic, with an increase of 442 − 77,402 cases per day [19]. During the investigation, China implemented a dynamic “Zero-COVID” policy, taking prompt actions to contain the outbreak of COVID-19 in the local area [20]. The specific measures include medically lockdown those who have had close contact with confirmed cases; large-scale nucleic acid testing; citywide home quarantine; the use of electronic health codes when entering public places; travel restrictions; and advocating for mask-wearing in public spaces. In certain situations, staff will remind individuals to wear masks or they will be prohibited from entering. During the policy implementation, China rigorously enforced the policy, and the policy was well implemented [21, 22].

The survey used Equal-probability sampling and non-equal-probability sampling. Equal-probability sampling (stratified sampling) at the provincial, municipal, district, township/subdistrict, and community/village levels (stratified sampling) and non-equal-probability sampling (quota sampling) at the community/village to individual level At least one surveyor or a panel of surveyors were recruited in each city. Investigators set up questionnaire points at health service centers or relevant health service stations in the sampling communities under their responsibility to conduct face-to-face surveys. And if they cannot conduct them due to the epidemic, the user uses the Online Questionnaire Star platform (https://www.wjx.cn/) to distribute the electronic questionnaire to each person to collect data. All participants obtained the participating respondent and record the questionnaire number issued to that person. Subjects were included in the study if they were ≥ 12 years old, provided written informed consent, and volunteered to participate in the study. A total of 23,414 questionnaires were collected, and after identifying and removing duplicate values, missing values and outliers with logical problems, a final sample of 21,916 was obtained, with a valid response rate of 93.6%.

### Variables

#### Characteristic variable

The characteristic variable in this study included respondents’ basic information (age stage, gender, education, chronic and current work status), family characteristics (family income) and personal health status (chronic), COVID-19 related (COVID-19 impact of lockdown on livelihoods, lockdown), Negative events, Smoking status (length of smoking), exposure to secondhand smoke (acceptation degree of passive smoking, acquaintance smoking, smoker smoked around, and stay in smoking area). See Supplementary Table S1 for details of definitions and classifications.

#### Self-efficacy

Self-efficacy was measured using the New General Self-Efficacy Scale short form (NGSES-SF3) [23]. The scale consists of 3 items, with a total score ranging from 0 to 12 points. See Table S2 in the Supplementary Material for details. In this study, the Cronbach coefficient for the NGSES-SF3 was 0.925.

#### Depression

Depression was measured using the Patient Health Questionnaire-9 (PHQ-9) [24]. It is a nine-item self-report scale developed to assess symptoms of depression. The items were rated on a scale of 0–3 (not done = 0), and the total scale score ranges from 0 to 27. Symptom severity can be illustrated through the total score, where 0–4 points are without depression; 5–9 points for mild depression; 10–14 points for moderate depression; 15–19 points for more severe depression; 20–27 points for severe depression. See Table S3 in the Supplementary Material for details.

#### Anxiety

Anxiety was measured using the Generalized Anxiety Disorder Questionnaire (GAD-7) developed by Robert L Spitzer [25]. The scale consists of seven items with Table S4 good reliability, as well as the validity of criteria, constructs, factors, and procedures. The cut-off point for optimal sensitivity (89%) and specificity (82%) was identified. See in the Supplementary Material for details.

#### Perceived social support

Social support was measured using the Perceived Social Support Scale short form (PSSS-SF3) based on the Zimet Perceived Social Support Scale [21]. A 3-item scale is divided into three dimensions: family support, friend support, and other supports, as shown in Table S5 in the Supplementary Material. These three items were rated on a scale of 1–7 (Strongly disagree = 1), with higher scores indicating a greater perception of social support. In this study, the Cronbach coefficient for the PSSS-SF3 was 0.943.

### Statistical analysis

First, we used EmpowerStats for descriptive analysis. Continuous variables were presented as mean ± standard deviation and categorical variables were reported as frequency N (%) and assessed using t-tests for continuous variables and chi-square tests for categorical variables. Next, we conducted univariate and multivariate logistic regression using Stata version 16.0 to examine the relationships between each potential predictor (demographic characteristics, perceived social support, depression, anxiety, and self-efficacy) and smoking outcome. Than, based on the results of the multivariate logistic regression, variables related to smoking behavior were included and a CART analysis was performed using R to identify high-risk populations for increased smoking behavior during COVID-19 and the factors that most deeply influenced the increase in smoking behavior.

Finally, we used R to evaluate the performance of CART. We calculated sensitivity, specificity, positive predictive value, negative predictive value, and accuracy of the CART model. In this study, sensitivity refers to the probability of correctly predicting smokers as smokers. Specificity refers to the probability of not predicting non-smokers as smokers. Positive predictive value represents the proportion of true smokers among the sample units predicted as smokers. Negative predictive value represents the proportion of true non-smokers among the sample units predicted as non-smokers. Accuracy refers to the proportion of correctly categorized smokers and non-smokers out of the total.

## Results

### Sample characteristics

A total of 21,916 valid data were collected. Table 1 showed the demographic, self-efficacy, anxiety, and perceived social support characteristics of the smoking and non-smoking population (P < 0.01). Compared to non-smokers, smokers are more anxious, more depressed, less accepting of secondhand smoke, experience more negative events, have more acquaintances smoking in front of them, perceive less social support, and have lower self-efficacy (P < 0.001). Both groups were predominantly aged 18–59 years, without chronic disease and COVID-19 lockdown measures (P < 0.01) (Table 1).

**Table 1:** **Descriptive analysis of sample characteristics on smokers and un-smokers. (mean ± SD)**

	Un-smokers	Smokers	P-value
N	18,658	3258	
COVID-19 impact of lockdown on livelihoods	60.5 ± 26.5	60.3 ± 28.1	0.690
Anxiety	4.6 ± 4.6	5.3 ± 5.0	< 0.001
Acceptation degree of passive smoking	14.7 ± 4.8	12.5 ± 5.0	< 0.001
Negative events	0.7 ± 1.1	0.9 ± 1.2	< 0.001
Perceived social support	15.2 ± 3.7	14.2 ± 4.2	< 0.001
Self-efficacy	7.9 ± 2.4	7.4 ± 2.7	< 0.001
**Gender**			
Male	8088 (43.3%)	2870 (88.1%)	< 0.001
Female	10,570 (56.7%)	388 (11.9%)	
**Age stage(year)**			
12 ~ 17	1954 (10.5%)	118 (3.6%)	< 0.001
18 ~ 59	13,242 (71.0%)	2405 (73.8%)	
> 60	3462 (18.6%)	735 (22.6%)	
**Current work status**			
Working	6198 (33.2%)	1403 (43.1%)	< 0.001
Student	6147 (32.9%)	433 (13.3%)	
Retired	2263 (12.1%)	493 (15.1%)	
Freelance	1985 (10.6%)	624 (19.2%)	
Unemployed	192 (1.0%)	50 (1.5%)	
Non-working	1873 (10.0%)	255 (7.8%)	
**Education**			
Primary and below	2800 (15.0%)	612 (18.8%)	< 0.001
Junior to senior secondary	9392 (50.3%)	1858 (57.0%)	
Tertiary and above	6466 (34.7%)	788 (24.2%)	
**Chronic**			
No	14,352 (76.9%)	2104 (64.6%)	< 0.001
Yes	4306 (23.1%)	1154 (35.4%)	
**Family income**			
< 3000	6060 (32.5%)	1169 (35.9%)	< 0.001
3001 ~ 5000	5749 (30.8%)	906 (27.8%)	
> 5000	6849 (36.7%)	1183 (36.3%)	
**Lockdown**			
No	17,404 (93.3%)	2972 (91.2%)	< 0.001
Yes	1254 (6.7%)	286 (8.8%)	
**Depression**			
Without depression	8101 (43.4%)	1197 (36.7%)	< 0.001
Mild depression	6465 (34.7%)	1164 (35.7%)	
Moderate depression	2529 (13.6%)	502 (15.4%)	
More severe depression	1161 (6.2%)	269 (8.3%)	
Severe depression	402 (2.2%)	126 (3.9%)	
**Length of smoking(year)**			
< 10	223 (29.5%)	1121 (34.4%)	< 0.001
10 ~ 20	171 (22.6%)	930 (28.5%)	
21 ~ 30	123 (16.2%)	661 (20.3%)	
31 ~ 40	119 (15.7%)	285 (8.7%)	
> 40	121 (16.0%)	261 (8.0%)	
**Acquaintance smoking**			
No	16,079 (86.2%)	2349 (72.1%)	< 0.001
Yes	2579 (13.8%)	909 (27.9%)	
**Smoker smoked around**			
Yes	8239 (77.1%)	2520 (90.2%)	< 0.001
No	2447 (22.9%)	274 (9.8%)	
**Stay in smoking area(day)**			
0	2274 (23.9%)	315 (13.5%)	< 0.001
1 ~ 4	3866 (40.6%)	997 (42.8%)	
5 ~ 7	3373 (35.5%)	1015 (43.6%)	

### Univariate logistic regression analysis and multivariate logistic regression analysis

Univariate regression analysis showed that the COVID-19 Impact of lockdown on Livelihoods was not associated with smoking behavior during COVID-19 (P = 0.246) (Table 2). Multivariate logistic regression analysis showed that having a chronic disease, higher perceived social support, lower self-efficacy, 31–40 years of smoking, absence of acquaintance smoking in front of them, staying in the smoking area, and lower acceptance of secondhand smoke were associated with the rise of smoking behavior during COVID-19 (P < 0.05) (Table 3).

**Table 2:** Univariate logistic regression analysis of smoking behavior change in COVID-19

	OR (95% CI)	SE	P-value
COVID-19 impact of lockdown on livelihoods	1.000 (0.998, 1.001)	0.000	0.690
Anxiety	1.032 (1.024, 1.040)	0.004	0.000
Acceptation degree of passive smoking	0.917 (0.910, 0.923)	0.003	< 0.001
Negative events	1.132 (1.098, 1.167)	0.018	< 0.001
Perceived social support	0.937 (0.928, 0.946)	0.006	< 0.001
Self-efficacy	0.925 (0.912, 0.939)	0.007	< 0.001
**Gender**			
Female	0.103 (0.093, 0.116)	0.005	< 0.001
**Age stage**			
18 ~ 59	3.007 (2.485, 3.640)	0.293	< 0.001
> 60	3.516 (2.872, 4.303)	0.363	< 0.001
**Current work status**			
Student	0.311 (0.278, 0.349)	0.018	< 0.001
Retired	0.962 (0.859, 1.078)	0.057	0.508
Freelance	1.389 (1.248, 1.546)	0.076	0.000
Unemployed	1.150 (0.838, 1.579)	0.186	0.386
Non-working	0.601(0.521, 0.694)	0.044	< 0.001
**Education**			
Junior to senior secondary	0.905 (0.818, 1.000)	0.046	0.052
Tertiary and above	0.558 (0.497, 0.625)	0.032	< 0.001
**Chronic**			
Yes	1.828 (1.688, 1.979)	0.074	< 0.001
**Family income**			
3001 ~ 5000	0.817 (0.744, 0.897)	0.039	< 0.001
> 5000	0.895 (0.820, 0.978)	0.040	0.014
**Lockdown**			
Yes	1.336 (1.168, 1.527)	0.091	< 0.001
**Depression**			
Mild depression	1.219 (1.117, 1.329)	0.054	< 0.001
Moderate depression	1.343 (1.199, 1.505)	0.078	< 0.001
More severe depression	1.568 (1.355, 1.814)	0.117	< 0.001
Severe depression	2.121 (1.721, 2.615)	0.226	< 0.001
**Length of smoking(year)**			
10 ~ 20	1.082 (0.871, 1.345)	0.120	0.478
21 ~ 30	1.069 (0.841, 1.359)	0.131	0.586
31 ~ 40	0.476 (0.368, 0.616)	0.063	< 0.001
> 40	0.429 (4.354, 5.804)	0.057	< 0.001
**Acquaintance smoking**			
Yes	2.413 (2.211, 2.632)	0.107	< 0.001
**Smoker smoked around**			
Yes	2.732 (2.392, 3.119)	0.185	< 0.001
**Stay in smoking area(day)**			
1 ~ 4	1.862 (1.624, 2.135)	0.130	< 0.001
5 ~ 7	2.172 (1.894, 2.492)	0.152	< 0.001

*Note*: OR (95% CI): Odd Ratio (95% Conf. Interval)

**Table 3:** Multivariate logistic regression analysis of smoking behavior change in COVID-19

	OR (95% CI)	SE	P-value
Anxiety	1.000 (0.966, 1.035)	0.018	0.988
Acceptation degree of passive smoking	0.956 (0.934, 0.978)	0.011	<0.001
Negative events	1.030 (0.944, 1.123)	0.047	0.512
Perceived social support	1.039 (1.003, 1.075)	0.018	0.031
Self-efficacy	0.900 (0.851, 0.949)	0.025	<0.001
**Gender**			
Female	0.895 (0.654, 1.223)	0.143	0.486
**Age stage**			
18 ~ 59	0.859 (0.500, 1.475)	0.237	0.582
> 60	0.726 (0.378, 1.395)	0.242	0.337
**Current work status**			
Student	0.706 (0.508, 0.980)	0.118	0.037
Retired	0.804 (0.525, 1.231)	0.175	0.316
Freelance	1.006 (0.734, 1.379)	0.162	0.969
Unemployed	2.266 (0.748, 6.868)	1.282	0.148
Non-working	0.905 (0.574, 1.426)	0.210	0.667
**Education**			
Junior to senior secondary	0.859 (0.616, 1.197)	0.145	0.369
Tertiary and above	0.780 (0.524, 1.160)	0.158	0.219
**Chronic**			
Yes	0.502 (0.402, 0.628)	0.057	<0.001
**Family income**			
3001 ~ 5000	1.072 (0.822, 1.397)	0.145	0.608
> 5000	1.062 (0.824, 1.370)	0.138	0.642
**Lockdown**			
Yes	0.819 (0.580, 1.156)	0.144	0.256
**Depression**			
Mild depression	1.014 (0.763, 1.349)	0.148	0.921
Moderate depression	0.953 (0.644, 1.410)	0.190	0.809
More severe depression	0.869 (0.519, 1.456)	0.229	0.593
Severe depression	1.931 (0.871, 4.279)	0.784	0.105
**Length of smoking**			
10 ~ 20	1.200 (0.907, 1.587)	0.171	0.201
21 ~ 30	1.180 (0.847, 1.643)	0.199	0.328
31 ~ 40	0.576 (0.407, 0.817)	0.103	0.002
> 40	0.870 (0.563, 1.342)	0.193	0.528
**Acquaintance smoking**			
Yes	0.806 (0.644, 1.006)	0.091	0.057
**Smoker smoked around**			
Yes	2.516 (1.897, 3.336)	0.362	<0.001
**Stay in smoking area(day)**			
1 ~ 4	1.371 (1.044, 1.801)	0.191	0.023
5 ~ 7	2.075 (1.540, 2.794)	0.315	<0.001

*Note*: OR (95% CI): Odd Ratio (95% Conf. Interval)

### Classification and regression tree (CART) analysis

**Fig. 1:**
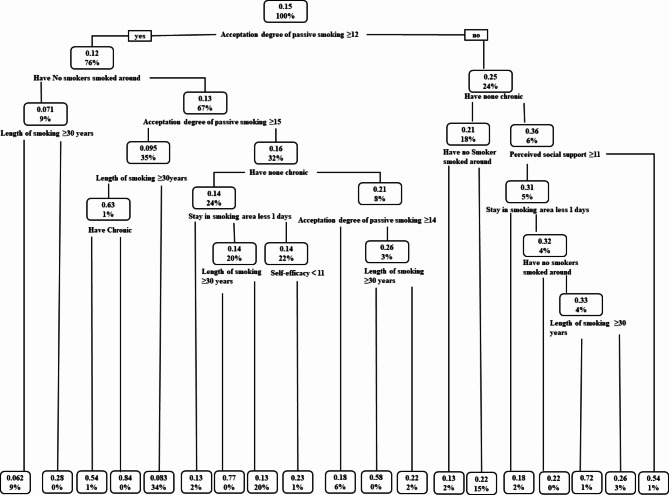
Classification and regression tree analysis of factors influencing smoking behavior

The CART analysis (Fig. 1) used a sample of smoking individuals and identified attitudes toward secondhand smoke as the strongest predictor. The 100% in the parent node of Fig. 1 represents the entire smoking population in this study, while the 0.15 represents the 15% of the overall population included in the study. The first branch divided the smoking population into those with an acceptation degree of passive smoking ≥ 12 (76%) and those with a degree < 12 (24%). The branch for those with a degree ≥ 12 and with no smoker smoked around (72%) led to a subgroup with a length of smoking of 30 years or more, accounting for 28% of the total smoking population. The branch for those with a score < 12 were non-chronic disease patients.

The branch for those with an acceptation degree of passive smoking of ≥ 12 indicates that no smokers smoked around. Among those with an acceptation degree of passive smoking of ≥ 12, have no smokers smoked around, and a length of smoking of ≥ 30 years, a subgroup leads to a terminal node, accounting for 28% of the total smoking population. This branching process is repeated until the sample is classified into 15 risk profiles (bottom row of Fig. 1). Currently, the subgroup with a high acceptation degree of passive smoking, have no smokers smoked around, and a length of smoking of ≥ 30 years is identified as the highest smoking risk (34%).

### Performance evaluation of classification and regression tree (CART)

In this study, the CART model demonstrated high specificity (99%), high positive predictive value (71%), high negative predictive value (88%), and a high accuracy rate (87%). But sensitivity (20%) is low, it may be due to category imbalance that large sample size gap between smokers and non-smokers in this study (Table 4).

**Table 4:** Performance of classification and regression tree (CART)

Statistic	
Sensitivity	20%
Specificity	99%
Positive predictive value	71%
Negative Predictive Value	88%
Accuracy	87%

## Discussion

Smoking is a closely related factor to COVID-19, and controlling smoking behavior is of great significance for the prevention and treatment of COVID-19. Identifying high-risk subgroups for smoking during the pandemic can enable targeted prevention and effective reduction in smoking behavior. Therefore, this study used CART analysis to identify high-risk subgroups for smoking behavior during COVID-19 and determine the factors that have the deepest influence.

Lockdown is an important factor influencing smoking behavior during COVID-19. It refers to the measures taken by various countries to prevent the spread of COVID-19, such as home quarantine, closure of entertainment venues, and isolation and quarantine measures [26]. Due to the differences in social background and lockdown measures, the impact of lockdown on smoking behavior may also vary [26]. In this study, lockdown mainly refers to three measures: home quarantine, activities within the community, and activities within the city. Previous studies have shown that during lockdown, stress and depression increase, and people tend to smoke more frequently, while the number of people attempting to quit smoking decreases [27]. However, the results of this study suggest that lockdown is associated with a decrease in smoking behavior. This may be due to the inability to purchase cigarettes during the home quarantine period [7] and an increase in motivation to quit smoking due to an increased perception of the harm of COVID-19 [28].

According to the CART model, currently, the subgroup with a high acceptation degree of passive smoking, have no smoker smoked around them, and a length of smoked of 30 years or more has the highest smoking rate during the COVID-19 pandemic. The acceptation degree of passive smoking is the main determinant of smoking behavior during the pandemic. This may be because people are more attentive to personal health protection during the COVID-19 pandemic and are more sensitive to the perceived harmfulness of tobacco [29], which may lead to a lower acceptance of secondhand smoke [30], resulting in a reduction in smoking behavior.

According to the CART model, during the COVID-19 pandemic, people are more likely to smoke when they are in the presence of have no smoker smoked around, which is contrary to previous research results. Previous studies have shown that individuals are more likely to start smoking when family and friends around them smoke [31, 32]. This may be due to an increase in personal protection awareness during the COVID-19 pandemic. As COVID-19 primarily affects the respiratory system, wearing a mask is an important preventive measure against COVID-19 [33]. During the period of this study, China was still experiencing the peak of the COVID-19 pandemic [19]. Despite the presence of individual variations, due to the Chinese government’s advocacy for mask usage and the concurrent increase in public health awareness among the population, there is a high level of acceptance and compliance with mask-wearing during the COVID-19 pandemic [34]. Even in 2023, when the COVID-19 pandemic has largely subsided, residents continue to exhibit good mask-wearing habits [35]. When people remove their masks to smoke, others may become more attentive to wearing masks due to fear of contracting COVID-19. Thus, when people are not smoking around them, individuals may be more likely to smoke. This conclusion needs to be verified in other countries. This result is opposite to our Logistic regression results, which may be due to CART examining the interaction between variables, which is why CART is widely used in exploring risk factors [36, 37]. Additionally, due to nicotine dependence, those with a longer smoking history have stronger nicotine dependence and more severe withdrawal symptoms, making it harder for them to reduce smoking behavior [38]. Therefore, our study shows that individuals with a length of smoking of 30 years or more are more likely to smoke during the COVID-19 pandemic. The group with a length of smoking of 40 years or more is not significant in the multiple regression results but is included in the CART model. There are two possible reasons for this. On the one hand, CART has greater resistance to multicollinearity compared to other parametric methods [36, 37]. On the other hand, CART is a decision tree model that only considers which variables can better predict the increase in smoking behavior during the COVID-19 pandemic and form the best classification, without considering variable significance issues.

Having a chronic illness is also a significant predictor, as non-chronically ill individuals are more likely to smoke. Smoking is strongly associated with chronic diseases [39], and China’s disease spectrum has shifted towards chronic, non-communicable diseases [40]. Additionally, chronic illness patients have a higher severe disease rate after contracting COVID-19 [41, 42]. To reduce the harm of chronic diseases, doctors are more likely to advise chronic illness patients to quit smoking, and patients are also more likely to accept smoking cessation advice from doctors [43, 44].

Regarding these issues, first, more attention should be paid to long-term smokers, and more specialized smoking cessation help should be provided to them. For example, the smoking cessation clinic actively promoted in China is an effective method [45]. Secondly, for individuals who are more exposed to secondhand smoke, tobacco education should be strengthened to enhance awareness of the hazards of secondhand smoke. Moreover, due to the requirement to wear masks in public areas during the COVID-19 period, smoking behavior has also been reduced. Therefore, during the COVID-19 pandemic, the “mask protection” effect can be fully utilized to guide smoking cessation behavior. Even if individuals around them are not smoking, environmental smoke may still carry and spread the virus, so it is necessary to wear masks and avoid smoking. Finally, doctors and non-chronically ill patients should also raise awareness of smoking cessation. Tobacco causes great harm to human health, and doctors’ smoking cessation advice is feasible in promoting patient smoking cessation [46], making doctors an important candidate in promoting smoking cessation, and doctors should also actively provide smoking cessation help to non-chronically ill patients.

Finally, this study explored the high-risk groups for smoking, and future studies should also delve deeper into the triggers for smoking cessation to provide a guiding direction for tobacco control policies and to form a continuity study to enrich policy guidelines.

### Strength and limitation

Our study conducted a national survey using quota sampling, which can balance differences between regions and reflect the situation nationwide. Secondly, we focused on smoking behavior during the COVID-19 period and comprehensively analyzed the factors that influence smoking behavior in the context of epidemic prevention and control. Finally, our study results further revealed the mutual interactions between the most important risk factors and other influencing factors, thus identifying the high-risk group for smoking during the COVID-19 period.

However, this study also has some limitations. First, the study is a cross-sectional survey and does not establish causal relationships. Second, there may be other risk factors that affect smoking behavior during the COVID-19 period that were not included in this study. Finally, the sensitivity of CART in this study was relatively low, probably because of the small number of smokers in this study, which was large sample size gap between smokers and non-smokers. But even so, the accuracy of CART was high.

## Conclusion

In general, this study was based on a national sample and used CART analysis to explore the high-risk population for increased smoking behavior during the COVID-19 period. The results showed that people with a high acceptation degree of passive smoking, have no smokers smoked around, and a length of smoking of ≥ 30 years were the subgroups with the highest smoking behavior during the COVID-19 period. Acceptation degree of passive smoking was the strongest predictor of smoking behavior during the COVID-19 period. It is important to pay more attention to long-term smokers and non-chronic disease patients, raise awareness of the hazards of smoking and secondhand smoke, and take advantage of the “mask effect” during the epidemic period to reduce smoking behavior during the COVID-19 pandemic.

### Electronic supplementary material

Below is the link to the electronic supplementary material.

Supplementary Material 1: Variable description, NGSES-SF3, PHQ-9, GAD-7, PSSS-SF3 and assignment criteria

Supplementary Material 2: The R package of classification and regression tree (CART)

## Data Availability

All data generated or analyzed during this study are with the corresponding author. She is available to take any questions about the datasets. Persons who have made outstanding contributions or assisted in this study may apply for the use of the data only after submitting the study hypothesis and signing a data confidentiality agreement. There is no fee for the data opening plan. Publication of the study results will include processed data only, and personal information will remain anonymous.

## Declarations

Classification and Regression Tree (CART).

New General Self-Efficacy Scale short form-3 (NGSES-SF3).

Patient Health Questionnaire-9 (PHQ-9).

Generalized Anxiety Disorder Questionnaire (GAD).

Perceived Social Support Scale short form (PSSS-SF3).
