# Supplementary material for: Risk profiles for smoke behavior in COVID-19: a classification and regression tree analysis approach
Source: BMC Public Health. 2023 Nov 21;23:2302. doi: 10.1186/s12889-023-17224-z (PMC10664606; doi:10.1186/s12889-023-17224-z)
Supplement: Supplementary file 1 — Supplementary Material 1: Variable description, NGSES-SF3, PHQ-9, GAD-7, PSSS-SF3 and assignment criteria [file 12889_2023_17224_MOESM1_ESM.docx]

**Supplementary Table S1 Variable Description.**

| Variable name | Definition or code |
| --- | --- |
| COVID-19 Impact of lockdown on Livelihoods | The respondents fill in by themselves, and the assignment range is 0~100. |
| Acceptation degree of passive smoking | Total score range 0-21; the higher the score the higher the level of acceptance. |
| Negative events | It is defined as whether you have experienced a negative life event in the last year. 0=no; 1=yes |
| Gender | 1=male; 2=female. |
| Age stage(year) | 1=12~17; 2=18~59; 3=60~ |
| Current work status | 1=working; 2=student; 3=retired; 5=freelance; 5=unemployed; 6=non-working. |
| Education | 1=primary and below; 2=junior to senior secondary; 3=tertiary and above. |
| Chronic | Defined as whether or not them have the chronic condition. 0=no; 1=yes |
| Family income | It is defined as the per capita monthly income of a household. 1=<3000; 2=3001~5000; 3=>5000 |
| Lockdown | Defined as whether or not a lockdown has been imposed because of COVID-19. 0=no; 1=yes |
| Length of smoking | It is defined as the time from the complete first cigarette smoking to the present, in years.  0=<10; 1=10~20; 2=21~30; 3=31~40; 4=>40 |
| Acquaintance smoking | It is defined as whether there are acquaintances around who smoke.  0=no; 1=yes |
| Smoker smoked around | It is defined as whether the smoker will smoke in person. 0=no; 1=yes |
| Stay in smoking area | It is defined as the amount of time spent in the smoking area in the last seven days, in days.  0=0  1=1~4  2=5~7 |

**Supplementary Table S2 New General Self-Efficacy Scale short form (NGSES-SF3) and Assignment Criteria.**

There are 3 sentences below, each followed by 5 answers. Please choose one answer after each sentence according to your actual situation.

Strongly disagree =0; Disagree =1; Neither agree nor disagree =2; Agree =3; Strongly agree =4

Total score ranging from 0 to 12 points, the higher the score, the better the self-efficacy.

| 1.When facing difficult tasks, I am certain that I will accomplish them. |
| --- |
| 2.I will be able to successfully overcome many challenges. |
| 3.I am confident that I can perform effectively on many different tasks. |

**Supplementary Table S3 Patient Health Questionnaire-9 (PHQ-9) and Assignment Criteria.**

In the past two weeks, have you often been troubled by the following problems. Please choose one answer after each sentence according to your actual situation.

Never =0; A few days =1; Just over half a day =2; Nearly every day =3

The score of 0-4 points are without depression; 5-9 points are mild depression; 10-14 points are moderate depression; 15-19 points are more severe depression; 20-27 points are severe depression.

| 1. Little interest or pleasure in doing things. |
| --- |
| 2. Feeling down, depressed, or hopeless. |
| 3. Trouble falling or staying asleep, or sleeping too much. |
| 4. Feeling tired or having little energy |
| 5. Poor appetite or overeating |
| 6. Feeling bad about yourself—or that you are a failure or have let yourself or your family down |
| 7. Trouble concentrating on things, such as reading the newspaper or watching television |
| 8. Moving or speaking so slowly that other people could have noticed? Or the opposite—being so fidgety or restless that you have been moving around a lot more than usual |
| 9. Thoughts that you would be better off dead or of hurting yourself in some way |

**Supplementary Table S4 Generalized Anxiety Disorder Questionnaire (GAD-7) and Assignment Criteria.**

In the past two weeks, how often have you experienced the following symptoms in your life? Please choose one answer after each sentence according to your actual situation.

Not at all =0; Several days =1; More than half the days =2; Nearly every day =3

Total score range 0 to 21; 0-4 no GAD; 5-9 mild GAD; 10-14 moderate GAD; 15-21 severe GAD

| 1. Feeling nervous, anxious or on edge. |
| --- |
| 2.Not being able to stop or control worrying. |
| 3. Worrying too much about different things. |
| 4. Trouble relaxing. |
| 5. Being so restless that it is hard to sit still. |
| 6. Becoming easily annoyed or irritable. |
| 7. Feeling afraid as if something awful might happen. |

**Supplementary Table S5 Perceived Social Support Scale short form (****PSSS-SF3) and Assignment Criteria.**

Please choose an answer after each sentence based on how you feel socially supported in practice.

Strongly disagree =1; disagree=2; Slightly disagree =3; Neutral =4; Slightly agree =5; agree =6; Strongly agree =7

The total score ranges from 0 to 21; higher scores indicate higher levels of overall social support perceived by the individual

| 1. I was able to get emotional help and support from my family when I needed it. |
| --- |
| 2. I have friends who can really help me. |
| 3. There are certain people in my life (leaders, relatives, colleagues) who care about my feelings. |
